# Inherited anoxia tolerance and growth performance can result in enhanced invasiveness in hybrid fish

**DOI:** 10.1242/bio.060342

**Published:** 2024-10-21

**Authors:** Konrad Dabrowski, Remigiusz Panicz, Kevin J. Fisher, Boris Gomelsky, Piotr Eljasik

**Affiliations:** ^1^ School of Environment and Natural Resources, Ohio State University, Columbus, Ohio, 43210, USA; ^2^ Faculty of Food Sciences and Fisheries,West Pomeranian University of Technology, Szczecin, Szczecin, 71-065, Poland; ^3^ School of Aquaculture and Aquatic Sciences, Kentucky State University, Frankfort, Kentucky, 40601, USA

**Keywords:** Alcohol dehydrogenase, Cyprinid hybrid, Ethanol synthesis, Gene expression, Hypoxia

## Abstract

Northern hemisphere freshwater ecosystems are projected to experience significant warming and shortening of winter duration in this century. This change coupled with depletion of oxygen (hypoxia) will result in a shift toward fish species with higher optimal temperatures for growth and reproduction that can mitigate hypoxic stress. Here, we tested the assumption that reproduction between two distant species, i.e. anoxic-intolerant common carp (*Cyprinus carpio*) and anoxic-tolerant goldfish (*Carassius auratus*), results in the expression of genes responsible for ethanol synthesis (*alcohol dehydrogenase* and *pyruvate dehydrogenase subunit E1β_2_*). The expression of this ethanol-producing pyruvate decarboxylase pathway may transform the biochemical characteristics of progeny into anoxic-tolerant hybrids, expanding their suitable environmental range and potentially increasing invasiveness. Concurrently, a genetic strategy for improving fish tolerance to oxygen-depleted environments will be a valuable physiological trait in fish culture. Differential quantification of gene expression by analyzing mRNA revealed that, compared with koi×koi, koi female×goldfish male (F_1_ hybrid) possessed the *pyruvate dehydrogenase subunit E1β_2_* gene construct, which was expressed at significantly greater levels in red muscle. The potential of this hybrid to both survive in extreme anoxic conditions and grow at elevated water temperatures would likely contribute to their ecological success.

## Teaser

**Summary:** We provide evidence that a hybrid line of koi carp × goldfish is characterized by expression of mRNA from genes responsible for synthesis of ethanol and consequently for anoxia tolerance.

## INTRODUCTION

Blazka (1958) was the first investigator to describe anaerobic metabolism in fish of the genus *Carassius*. At low temperatures (5°C), compared with other teleost species, crucian carp (*Carassius carassius*) were found to tolerate anoxic conditions for a minimum of 2 months without excessive accumulation of lactic acid under anaerobic conditions. Piironen and Holopainen (1986) expanded on these findings by documenting that crucian carp tolerance to anoxia was maintained for 4.5 months at 3°C. However, these authors also noted that tolerance to anoxia was seasonally influenced with resistance being lost in spring as water temperature increased. Shoubridge and Hochachka (1980) addressed the mechanism of this unprecedented phenomenon in vertebrates with *Carassius* sp. acclimated for an unknown duration to 4°C. These authors discovered that while experiencing anoxic conditions at low water temperature, *Carassius* sp. can synthesize and excrete ethanol as the end product of glucose metabolism. These findings contradicted earlier assertions by Prosser et al. (1957) that the tolerance of goldfish acclimated to hypoxia (at 22°C) could be explained by increases in hemoglobin levels and lowered oxidative activity. By utilizing carbon monoxide, oxygen transport via the gills was blocked at poisonous concentrations, and a 32-fold increase in lactate levels was observed in whole-fish tissues from the anoxic goldfish *Carassius auratus* compared to the normoxic control (Shoubridge and Hochachka, 1980). This finding contrasts with the data of Blazka (1958), where complete anoxia was imposed for 6 h at 20°C by hydrogen sulfide with no oxygen debt being observed.

Fagernes et al. (2017) further expanded the understanding of anaerobic metabolism in crucian carp and goldfish by identifying two genes responsible for the ethanol-producing pyruvate decarboxylase pathway. The authors examined several tissues, including the brain and red and white muscle, in crucian carp following short anoxia and reoxygenation, identifying several pyruvate dehydrogenases that convert acetaldehyde to ethanol. In anoxic-intolerant species, such as common carp, these alcohol decarboxylase genes have not been found to be expressed at levels comparable to those observed in goldfish, and no ethanol excretion was found (Johnston and Bernard, 1983).

The production of hybrids of common carp, *Cyprinus carpio* female and goldfish males and their reciprocal crosses was described by Matsui (1933). Males of this hybrid cross were found to be sterile (Matsui, 1933), which can be explained by postzygotic reproductive isolation related to Haldane's rule, where hybrid sterility occurs less frequently in closely related species than in distant species (Russel, 2003). Moreover, female hybrids were found to produce viable triploid progeny when backcrossed to either parental species (Matsui, 1934). The offspring produced from backcrossing hybrid females with common carp were found to be triploid (Cherfas et al., 1981; Gomelsky et al., 2012) due to the production of diploid eggs in a high proportion of female fish (Gomelsky et al., 2012). A lack of fertile males and the production of only sterile triploid offspring by females are likely the reasons why this hybrid, which historically has been common in the Great Lakes (Taylor and Mahon, 1977; Trautman, 1981), has only sporadically established significant populations. However, significant warming of the Great Lakes (Farmer et al., 2015) is predicted in the present century, extending conditions closer to the thermal optimum for the growth and reproduction of common carp and goldfish. These increases in temperature may allow spawning to expand in both space and duration. For example, in the subtropical regions of Australia and Africa, common carp are exposed to extended durations of optimal temperatures for maturation (egg and sperm production) (Hailu, 2013; Smith and Walker, 2004). Davies et al. (1986), when examining effects of light and temperature regime on spawning carp in captivity, reported that completion of the ovarian cycle requires no less than 3 months in common carp. Since common carp reproductive plasticity allows for multiple spawning events during a year, there is increased potential to produce F_1_ hybrids between common carp and goldfish.

Balashov and Recoubratsky (2011) [the article referenced is an English translation from Russian, which has many inconsistencies from the original. The information cited here is based on the original Russian language version of this article, Balashov and Recoubratsky (2011). *Voprosy Ikhtiologii,* 51;665–669.] investigated the hypoxia tolerance of diploid hybrids of a female *C. auratus* (English translation called these silver crucian carp) and common carp male. These authors produced seven consecutive gynogenetic generations by activating diploid eggs of hybrid females with UV-irradiated common carp sperm. The hypoxia tolerance of these gynogens was compared with that of two groups of triploid backcross hybrids that differed in terms of the ratio of common carp to *C. auratus* haploid genomes. These authors found that the hypoxia tolerance of fish increased with an increasing proportion of *Carassius* heredity. The highest hypoxia tolerance was detected in backcross triploid hybrids with a dominant number of chromosomes of *C. auratus*. We do not know at this stage which chromosomes are present in the hybrid. Compared with those of common carp, survival of fish of this genotype increased more than 12-fold under hypoxia at 5°C (Balashov and Recoubratsky, 2011).

There are several potentially important implications of this extreme tolerance to anoxia (hypoxia) observed in hybrids of common carp and goldfish (*C. auratus*). First, the presence of this hybrid in lakes and ponds where overwintering conditions often lead to mass winterkills due to anoxia could result in this hybrid being found in higher proportions (60–90%) than common carp (Trautman, 1981). Second, anoxic-tolerant hybrids that inherit the increased growth potential of common carp, which is far superior to that of goldfish, can become staple aquaculture fish species in parts of the world (Southeast Asia), where cyprinid fishes are appreciated as sustainable, valuable, and healthy foods for human consumption.

Brauer et al. (2023) recently described an example of ecological niche overlap among five species of rainbowfish. Evidence of a high proportion of hybrids being present due to the predominance of a parental species with a wider tolerance range to environmental conditions was provided. They further examined the genome-environment association with climate variables and concluded that hybrids possessed a greater frequency of climate-linked alleles that allowed them to better handle the environmental variation associated with climate change (Brauer et al., 2023). Hybridization may also be responsible for the transfer of genes from one parental species to progeny, which results in the transmission of hypoxia tolerance.

We tested the hypothesis that the hybridization of two distantly related cyprinids, anoxic-intolerant common (koi) carp and anoxic-tolerant goldfish, would result in the transfer of the genes responsible for ethanol production to their progeny. We analyzed the expression of a key gene involved in growth and development, i.e. *insulin-like growth factor 1* (*igf-1*) and two genes involved in the ethanol production pathway, i.e. *alcohol dehydrogenase* (*adh*) and *pyruvate dehydrogenase subunit E1β_2_* (*pdh E1β_2_*) in brain, hepatopancreas, red muscle, and white muscle of purebred koi (♀K×♂K) and koi x goldfish hybrids (♀K×♂G). Additionally, this is the first investigation, to our knowledge, of the growth performance of half-siblings of purebred koi and koi x goldfish hybrids.

## RESULTS

### Experiment one

The nature of the interaction between cross (♀K×♂G and ♀K×♂K) and age (days post fertilization, dpf) was assessed for total length, and no evidence for a significant interaction effect (*P*=0.111) or main effect of cross (*P*=0.5196) was identified. However, there was evidence for a significant main effect of age (*P*=0.0033) (Fig. S1). Samples collected at 12 dpf were the lowest, with samples collected at 19 dpf and 34 dpf not significantly different from each other (Δ=16.0±0.8 mm, *P*=0.06). However, the total length of the fish was greatest at 48 dpf.

**Fig. 1.**
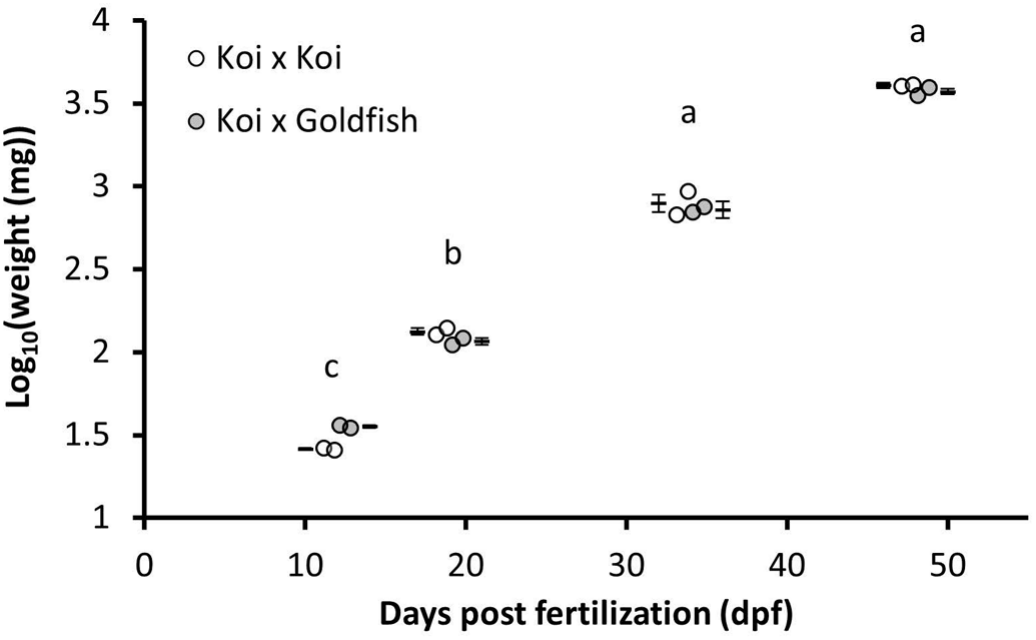
**Log_10_(weight (mg)) of koi x koi (**♀**K×**♂**K) and koi x goldfish hybrid (**♀**K×**♂**G) over the course of experiment one (12-48 dpf).** The data points represent replicate means (*n*=2) obtained from subsamples of *n*=10 fish per replicate, and the bars are lsmeans±SE. Repeated measurements analysis identified a significant main effect of age (*P*=0.008). A Tukey's adjustment was used and ages (dpf) not sharing common letters are significantly different (*P*<0.05).

No evidence of a significant interaction effect (*P*=0.153) or a main effect of cross (*P*=0.9889) on fish weight was identified. There was evidence for a significant main effect of age (*P*=0.008) (Fig. 1). The samples collected at 12 dpf had the lowest weight recorded. The weights of the fish collected at 34 dpf and 48 dpf were the highest and not significantly different from each other [Δ=0.7118±0.035 log_10_ (mg), *P*=0.0573].

### Gene expression

**Fig. 2.**
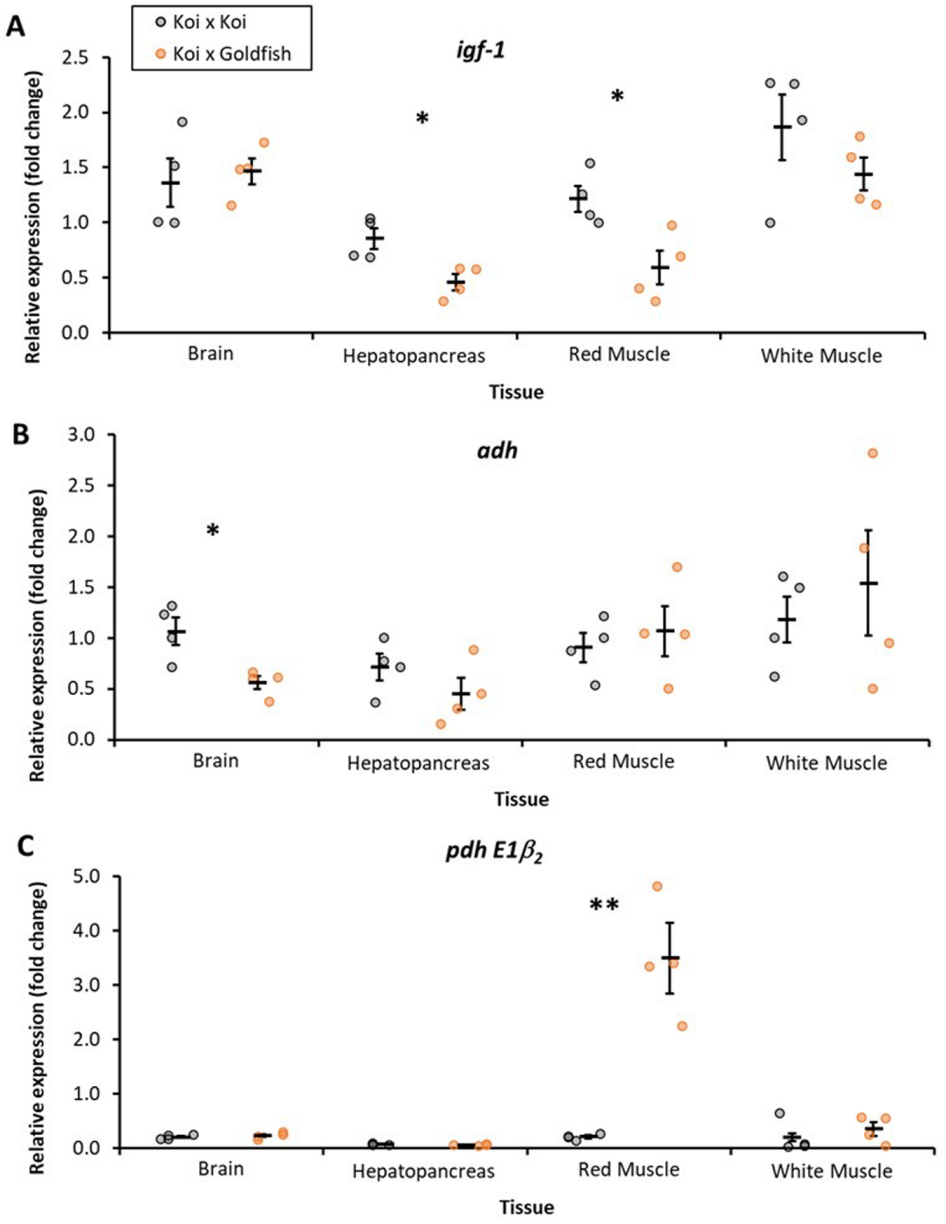
**Gene expression–relative expression (fold change) of (A) insulin-like growth factor 1 *igf-1* (B) alcohol dehydrogenase NADP-B *adh* (C) pyruvate dehydrogenase subunit *E1β_2_* in the brain, hepatopancreas, red muscle and white muscle of koi×koi (**♀**K×**♂**K) and koi x goldfish (**♀**K×**♂**G) hybrids.** The data points represent replicate measurements (*n*=4), and the bars are mean±s.e. Statistical tests were conducted on log transformed values. * indicates a significant difference at *P*≤0.05, ** indicates a significant difference at *P*≤0.01, between groups.

The effect of hybridization on the relative expression of three genes, *igf-1*, *adh*, and *pdh E1β_2_* (Fig. 2A,B,C), was investigated in the fish sampled at the conclusion of the first experiment. Expression of *igf-1* gene in the hepatopancreas (Fig. 2A) was significantly lower (*P*=0.01997) for ♀K×♂G (0.456±0.073-fold change) than for ♀K×♂K (0.854±0.095-fold change). Similarly, the expression of the *igf-1* gene in red muscle (Fig. 2A) was significantly lower (*P*=0.05) in ♀K×♂G (0.589±0.155-fold change) than in ♀K×♂K (1.214±0.120-fold change). There was no evidence for significant differences in the relative expression of *igf-1* gene in brain tissue (*P*=0.89) or in white muscle (*P*=0.35) between ♀K×♂G and ♀K×♂K (Fig. 2A).

The relative expression of the *adh* gene in brain tissue (Fig. 2B) was significantly lower (*P*=0.0157) for ♀K×♂G (0.563±0.064-fold change) than for ♀K×♂K (1.063±0.135-fold change). There was no evidence for significant differences in the relative expression of *adh* in the hepatopancreas (*P*=0.22), white muscle (*P*=0.78), or red muscle (*P*=0.71) between ♀K×♂G and ♀K×♂K (Fig. 2B).

The relative gene expression of *pdh E1β_2_* in red muscle (Fig. 2C) was significantly greater (*P*=0.0001) for ♀K×♂G (3.493±1.289-fold change) than for ♀K×♂K (0.207±0.053-fold change). There was no evidence for significant differences in the relative expression of *pdh E1β_2_* in brain tissue (*P*=0.56), in the hepatopancreas (*P*=0.38), or in white muscle (*P*=0.34) between ♀K×♂G and ♀K×♂K (Fig. 2C).

### Experiment two

Flow cytometry analysis of ten individuals from each cross confirmed that ♀K×♂G (c-value=1.75±0.03 pg) and ♀K×♂K (c-value=1.77±0.02 pg) were diploid as the expected c-value of koi carp is 1.70 pg (Tiersch et al., 1989) and goldfish is 1.77 pg (Vinogradov, 1998) with the hybrid ♀K×♂G expected to be in a similar range. The female koi carp×“F_2_ male koi×goldfish hybrid” (♀K×♂H) cross had eight individuals that were confirmed triploids (c-value=2.61±0.03 pg) and two diploids (c-value=1.79±0.01 pg), which were considered putative gynogens even though differences in phenotype were not apparent.

**Fig. 3.**
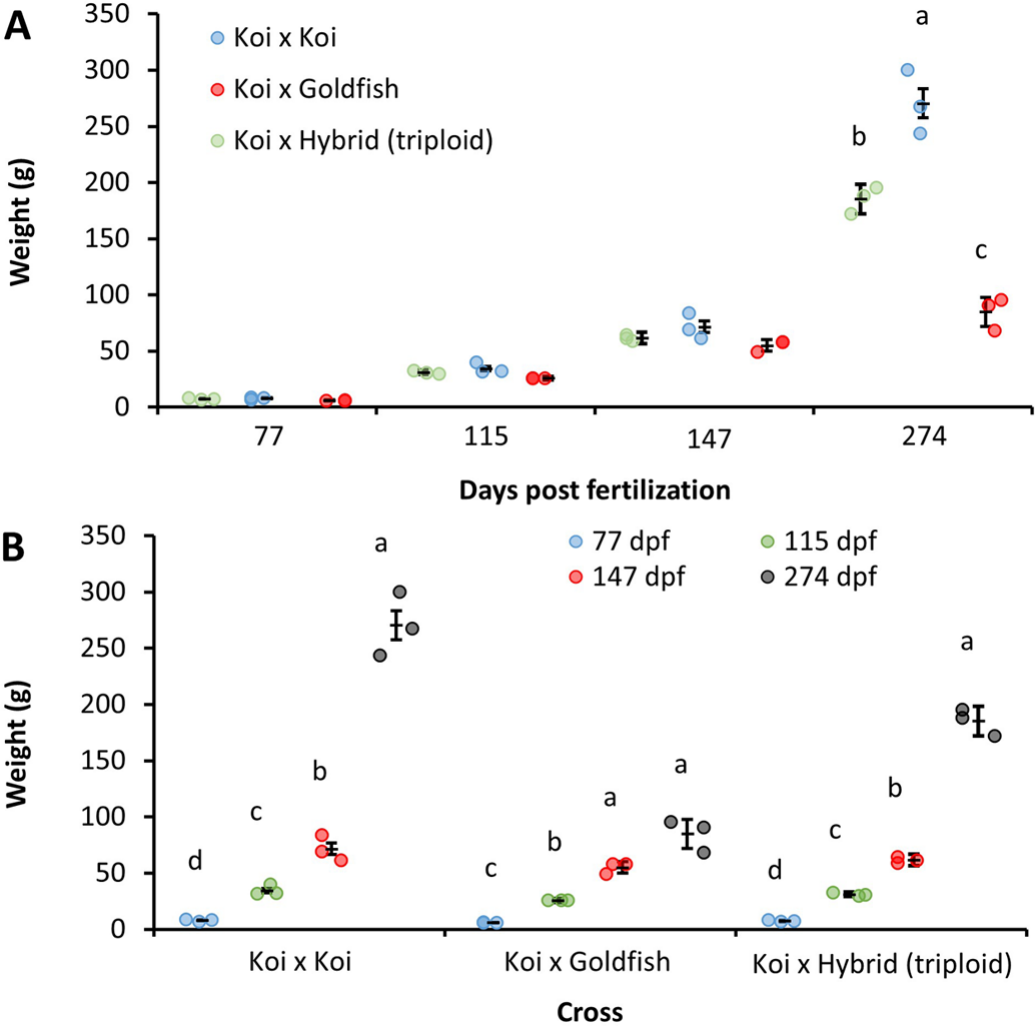
**Weight (mg) of koi×koi (**♀**K×**♂**K), koi x goldfish hybrid (**♀**K×**♂**G), and koi x hybrid (**♀**K×**♂**H) over the course of experiment two (77–274 dpf).** The data points represent replicate means (*n*=3) obtained from subsamples of *n*=10 fish per replicate, and the bars are lsmeans±SE. Repeated measurements analysis identified a significant interaction effect of cross and age (dpf) (*P*=0.0003). (A) Simple effects of cross within levels of age (dpf) are presented at the top and (B) simple effects of age (dpf) within levels of cross are presented on the bottom. A Bonferroni adjustment was utilized for multiple comparisons with levels not sharing a common letter being significantly different (*P*<0.05).

The effects of the interaction between cross (♀K×♂G, ♀K×♂K, and ♀K×♂H) and age (dpf) on total length (Fig. S2) and weight (Fig. 3) were investigated. A significant interaction effect was identified for both length (*P*=0.0295) and weight (*P*=0.0003). Simple effects of cross within the age range showed no significant differences (*P*>0.05) from 67 to 147 dpf, but a differential effect of cross was observed at 274 dpf for both total length and weight. At 274 dpf, ♀K×♂K had the highest recorded total length and weight, which were significantly different (Δ= 76.8±15.4 mm, *P*=0.0082; Δ= 185.6±16.0 g, *P*=0.0006) from those of ♀K×♂G, which was the lowest. ♀K×♂H total length was not significantly different from that of ♀K×♂G (Δ= 44.6±15.1 mm, *P*=0.0794) or ♀K×♂K (Δ=32.2±21.3 mm, *P*=0.1944). However, the weight of ♀K×♂H was significantly lower than that of ♀K×♂K (Δ=85.2±16.0 g, *P*=0.014) and significantly greater than that of ♀K×♂G (Δ= 100.4±16.1 g, *P*=0.0075). The simple effects of age within cross levels were all significantly different (*P*<0.05) for total length and weight. There was one exception for the weight of ♀K×♂G, which was not significantly different between 147 dpf and 274 dpf (Δ= 29.8±10.2 g, *P*=0.235).

**Fig. 4.**
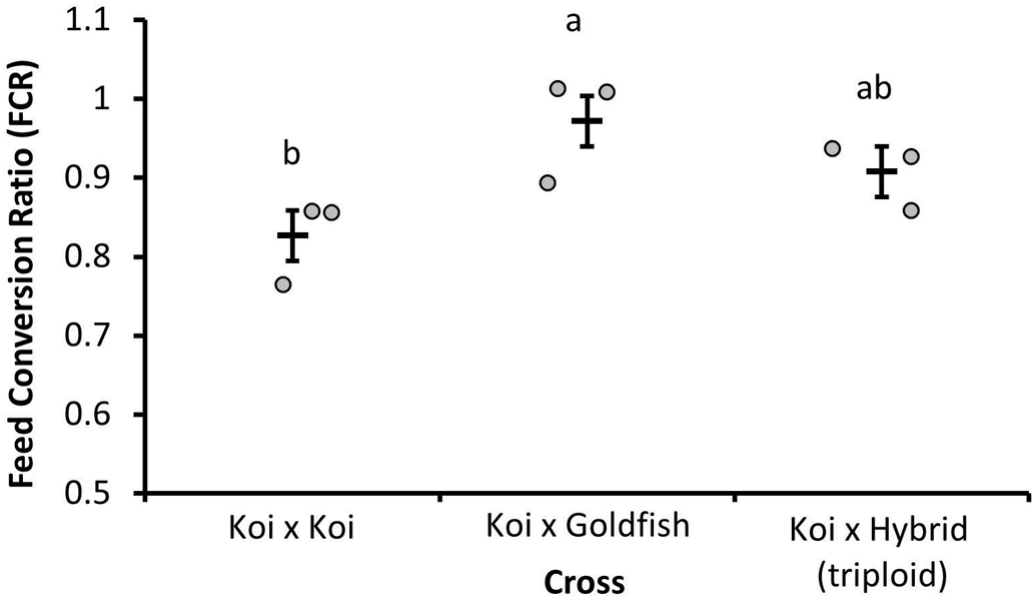
**Feed conversion ratio (FCR) of koi×koi (**♀**K×**♂**K), koi x goldfish hybrid (**♀**K×**♂**G), and koi x hybrid (**♀**K×**♂**H) over the period of 77–147 dpf of experiment two.** The data points represent replicate measurements (*n*=3), and the bars are lsmeans±s.e. A significant effect of cross was identified (*P*=0.049). Crosses with different letters are significantly different (*P*<0.05).

The influence of cross on the feed conversion ratio (FCR) over the 77–147 dpf period of experiment two was assessed (Fig. 4). A significant effect of cross was detected (*P*=0.049) in this period, with ♀K×♂G having the highest FCR (lowest feeding efficiency) and significantly different from ♀K×♂K (Δ=0.146±0.045, *P*=0.0417), which had the lowest FCR. The FCR of ♀K×♂H was not significantly different from that of ♀K×♂G (Δ=0.064±0.045, *P*=0.389) or ♀K×♂K (Δ=0.081±0.045, *P*=0.249).

## DISCUSSION

In a temperate climate with natural lake environments or common carp maintained for two winter periods in the conventional 3-year pond production cycle, this species is likely to experience hypoxic or nearly anoxic conditions. During this period, fish lose weight, and mortality may occur due to hypoxia (Billard, 1999). However, anoxic-tolerant *Carassius* sp. (Blazka, 1958; Dhillon et al., 2018) and hybrids of common carp and goldfish (Balashov and Recoubratsky, 2011) will be able to mitigate hypoxic stress.

During experiment one, differences in the growth rate of ♀K×♂K and ♀K×♂G first became apparent at 84 dpf (Fig. S3), when the mean weights of the ♀K×♂K and ♀K×♂G were 42.4±10.7 g and 51.7±9.1 g, respectively, with the hybrids being higher. This trend continued until ♀K×♂K began to grow faster than ♀K×♂G, when examined at 183 dpf, where the mean weights were 402.3±103.5 g and 266.4±74.8 g, respectively. ♀K×♂K continued to consistently grow at faster rates throughout, with the mean weights of ♀K×♂K and ♀K×♂G being 557±104 g and 288±96 g at 232 dpf, respectively (Fig. S3). This difference continued to increase through 365 dpf (1 year old), with ♀K×♂K and ♀K×♂G reaching 1053±282 g and 472±86 g, respectively (Fig. S3). These differences in growth occurred despite the lower rearing water temperature for ♀K×♂K than for ♀K×♂G (Fig. S4).

Differences in the growth rates of ♀K×♂K and ♀K×♂G in the second experiment became apparent later than were observed in experiment one at 274 dpf (Fig. 3) when the mean weights were 270±11 g and 85±11 g, respectively. No differences in weight were observed for ♀K×♂H between ♀K×♂K or ♀K×♂G, but the total length (Fig. S2) was significantly different, with ♀K×♂H being intermediate to the other crosses. Unlike in experiment one, fish from experiment two were housed in a single tank common garden design and were fed at a consistent rate throughout the grow-out period. This design likely resulted in interspecific competitive effects becoming apparent since feeding was restricted in this experiment. However, the fish also experienced identical thermal conditions throughout their growth.

For common carp, seasonal sums of water temperatures above 14°C highly correlate with pond fish biomass, whereas the effective temperature for growth appears to be between 19 and 25°C (Szumiec, 2005). The same author also concluded that the ‘upper thermal limit’ of common carp growth appears to be above 25°C even when environmental conditions deteriorate (hypoxia). Analysis of the differences in temperature profiles between koi carp and hybrids (Fig. S4) revealed that the preference for optimum koi carp growth might be lower than that for hybrids. The upper thermal limit for hybrid growth also appears to exceed 27°C even under deteriorated conditions of lowered (40% water saturation) oxygen levels. Although the present study does not directly compare the growth rate of ♀K×♂G and goldfish, the mean weight reported for 1-year-old *Carassius* sp. grown in ponds of 50–60 g (Kestemont, 1995) is severalfold lower than what was found for this ♀K×♂G at a comparable age. Tapkir et al. (2022) provides back calculated growth rate information for sister species *C. carassius* and *C. gibelio* in ponds, with both species being having much lower growth after 1 year, 54- and 64-mm standard length, respectively, than was observed for all crosses (184–261 mm total length at 274 dpf) in the current experiment (Fig. S2) even accounting for differences between measurement methods, standard versus total length. Additionally, they found that both *C. carassius* and *C. gibelio* reached a plateau for growth at 6–7 years at 90- and 120-mm standard body, respectively, (Tapkir et al., 2022) which is still less than what was observed in under a year for the current hybrids. While these aspects of hybrid koi x goldfish physiology appear to be potentially advantageous, further in-depth studies are needed.

In warm climates (southern China or Israel), common carp reach maturity in their first year, which may compromise growth rates (Hulata et al., 1974). However, one of the fastest growing common carp species (of European ancestry) in ponds was also described in Israel, where the mean weight at the age of 8 months was recorded to be 1.05–1.34 kg (Cherfas et al., 1994). Therefore, considering that this was the first generation of hybrids, growth must be considered satisfactory. The continued reproduction of hybrids through gynogenesis (using UV-irradiated sperm from common carp), sperm from sex-reversed ‘neomales’ of the XX genotype (Gomelsky et al., 1988), or extremely rare F_1_ fertile hybrid males (Delomas et al., 2017) (an exception to the Haldane rule) might result in an improved growth rate via selection (Balashov and Recoubratsky, 2011).

*igf-1* is a well-described hormone that is directly related to the growth, development, and reproduction of vertebrates, including fish (Renaville et al., 2002; Reinecke, 2010). The endocrine hormone *igf-1* is regulated and stimulated by growth hormone and is secreted mainly by hepatocytes and delivered via the blood to target tissues (Ohlsson et al., 2009). Furthermore, *igf-1* is expressed not only in the liver but also in several other organs where it likely has an autocrine/paracrine effect (Reinecke, 2010). We detected elevated *igf-1* expression in the brain and white and red muscle tissues in comparison to the hepatopancreatic *igf-1* expression in both koi×koi and goldfish hybrids (Fig. 2A). However, Beckman (2011) suggested that only the mRNA level of *igf-1* in the liver is correlated with the plasma *igf-1* level; thus, the *igf-1* level in the hepatopancreas should be considered only a growth index. Despite no evidence of differences in growth rate at 84 dpf, we found significant differences in hepatopancreatic *igf-1* mRNA levels between koi carp and goldfish hybrids (Fig. 2A). This could be partially explained by the fact that *igf-1* expression might change during the life of fish as well as be influenced by many environmental and physiological conditions (nutrition, temperature, sex, and maturity), which requires further study (Beckman, 2011).

The unique ability among fish to handle anoxic conditions has been found only in the genus *Carassius* and in the European bitterling *Rhodeus amarus* (Mourik et al., 1982; Shoubridge and Hochachka, 1980; Wissing and Zebe, 1988). The hypoxia-induced ethanol production pathway involves three key enzymes (lactate dehydrogenase, pyruvate dehydrogenase and alcohol dehydrogenase), which produce ethanol in red and white skeletal muscle, while other organs produce lactate during anoxia (Nilsson, 1988; Vornanen et al., 2009). In the present study, we assessed the activity of the *adh* in ♀K×♂K and ♀K×♂G, and we noted significantly greater *adh* expression in brain samples from ♀K×♂K than in those from ♀K×♂G (Fig. 2B). Dhillon et al. (2018) recently reported traces of activity of the *adh* enzyme in the red and white muscle tissues of seven non-*Carassius* cyprinid species, with notable *adh* activities in crucian carp (*C. carassius*) and goldfish that generated ethanol during anoxia. However, alcohol dehydrogenase activity in the hepatopancreas of *Carassius* sp. was severalfold lower than that in other cyprinids. Therefore, our finding of *adh* expression in ♀K×♂K should be associated with typical levels of vertebrate ethanol metabolism rather than with the response to hypoxia (Nilsson, 1988). The high expression of *pyruvate dehydrogenase E1β_2_* (Fig. 2C) in the red muscle of ♀K×♂K and ♀K×♂G (3.46-fold changes {2^−ΔΔCt^}) may indicate a significant increase in hypoxia tolerance at the molecular level in the 1st hybrid generation. Our results are similar to the data of Fagernes et al. (2017) for *C. carassius*, who identified that gene expression of the *pdh E1b2* subunit was higher in red muscles than in white. This difference arises from the fact that fish muscles comprise different types of fibers, each with distinct metabolic capabilities. Red muscles are responsible for slower and more sustained movements, primarily utilizing aerobic metabolism, whereas white muscles are adapted for quick, explosive movements, relying on anaerobic metabolism. Another similar aspect to the results published by Fagernes et al. (2017) is that activity of *pdh E1b2* subunit in both ♀K×♂K and ♀K×♂G were expressed at negligible levels in non-ethanol producing tissues, i.e. brain and hepatopancreas. Therefore, the higher activity of the *pdh E1b2* subunit found in our study could be linked to tolerance to hypoxic conditions, and from a molecular point of view, ♀K×♂G were hypoxia-tolerant. Similar results were obtained with respect to hypoxia tolerance in the 7th generation of gynogenetic goldfish (silver crucian carp) x common carp hybrids (Balashov and Recoubratsky, 2011). Although our study did not reveal that the ♀K×♂G outperformed the ♀K×♂K, the results of a gene expression analysis confirmed that intraspecies crossing led to significantly greater activity of *pdh E1β_2_* in ♀K×♂G hybrids. However, further trials are needed to confirm that the inheritance of anoxia tolerance in hybrids has been paternally inherited (goldfish) and in consequence increases ♀K×♂G ability to survive in oxygen-depleted environments, as was demonstrated in the gynogens of this reciprocal hybrid (Balashov, 2018). These trials are required to determine whether this new hybrid may translate this characteristic to enhanced invasiveness. Additionally, monitoring environmental conditions, conducting fish census surveys, and assessing gene expression analysis of *pdh* subunits in both purebred species of goldfish and common carp as well as their hybrids may provide important details for evaluating potential future shifts in dominant populations.

In salmonids, it has been described that during recombination of homologous chromosomes in hybrids, crossing over may occur, leading to the relocation of genes situated at some distance from the centromere or their elimination (Fujiwara et al., 1997; Pomianowski and Ocalewicz, 2021). Meiotic pairing configuration or endoreduplication can additionally occur during gametes formation and might result in sterility of one or both genders (Schmid et al., 2015; Arai, 2023) particularly in backcrosses when number of chromosomes differ between sperm and egg (hybrid cyprinids with 48 or 100 chromosomes; Dabrowski and Fisher, unpublished). Recombination of mitochondrial DNA (mtDNA) may also occur due to fusion of diploid sperm of hybrid cyprinid and *Carassius* egg (Guo et al., 2006). Despite the nearly same number of chromosomes in common carp and goldfish, microchromosomes have previously been identified in common carp that are absent in goldfish. Though, Raicu et al. (1972) was not able to confirm the presence of these microchromosomes in common carp from a European population. Therefore, in cyprinids like common carp and goldfish, which have similar numbers of chromosomes (98–104; Ohno et al., 1967; Raicu et al., 1972) the process of chromosome disjunction and potential elimination differs significantly from that observed in hybridized salmonids. Instead of chromosome elimination, there is a consistent formation of diploid gametes by somatic diploid individuals (Liu et al., 2008; Wang et al., 2019). The backcross of ♀K×♂G hybrids to koi carp or goldfish results in obtaining triploid individuals (Cherfas et al., 1981; Delomas et al., 2017; Warner et al., 2018), which was confirmed for ♀K×♂H in the current experiment. Therefore, the deletion of some specific chromosomes in cyprinid hybrids is much less likely than in salmonids.

Although we used koi carp females to cross with goldfish, it allows us to conclude that diploid gametes must have carried goldfish genes, including those responsible for the expression of anoxia-tolerant goldfish mtDNA. Based on results obtained from reciprocal crosses (Wang et al., 2019), somatic tetraploidy and diploid gametes in female common carp and goldfish in the F2 and F3 generations might become a more likely occurrence. In the reciprocal cross (female goldfish×male common carp), it is noteworthy that while all F1 hybrids produce diploid gametes, the F2 generation exhibits somatic diploidy. It is not until the F3 generation and beyond (F4–6) that tetraploid individuals are observed in progressively higher proportions in subsequent generations (Liu et al., 2008; Wang et al., 2019).

While the current experiment has confirmed the expression of goldfish mRNA in the hybrids, it does not necessarily indicate that the physiological trait of hypoxia tolerance has been conveyed to these hybrids. However, when this result is considered alongside the findings of Balashov and Recoubratsky (2011), which provided strong evidence of hypoxia tolerance at 20°C in these hybrids, a different picture emerges. Balashov and Recoubratsky (2011) demonstrated that the survival duration after reaching a critical dissolved oxygen level of 1.3 mg/L under joint trials exposure resulted in common carp surviving for a shorter duration (45 min) than that of the seventh generation gynogenetic (female) hybrids of goldfish and common carp (3 h). This suggests that these hybrids possess a physiological advantage in low oxygen environments compared to common carp.

## MATERIALS AND METHODS

Before the study began, all procedures were approved by the Ohio State University Institutional Animal Care and Use Committee (2008A0220-R3, 2008A0221-R3).

### Experimental design and feeding

The fish used in both experiments were produced at the Ohio State University Aquaculture Laboratory (Columbus, OH, USA) by fertilizing eggs from a single koi carp female with either a male koi carp, goldfish [originally obtained from a commercial supplier (Gomelsky et al., 2012)], or F_2_ koi×goldfish hybrid (Delomas et al., 2017). This is a significant improvement in experimental design compared to previous studies, as we solely introduced a half-sibling family effect into the analysis. Previous authors have used separate pools of female common carp to generate hybrids (with crucian carp) and purebred common carp (Skora and Erdmanski, 1985) for comparisons of progeny survival, growth performance, and fertility. By conducting experiments in this way, a female effect is added to the comparison that is often not accounted for in subsequent analyses. By utilizing a single pool of eggs/ovas that are fertilized with several different males, the need to consider differences in growth, specifically due to a female effect, is eliminated.

Two experiments were conducted to assess the growth of different crosses over time. The first experiment was conducted in 2019 and included the progeny of female koi×male koi (♀K×♂K) and female koi carp×male goldfish (♀K×♂G) for up to 1 year. This experiment was divided into two phases, with the first occurring from 5 to 47 dpf and the second occurring from 48 to 365 dpf. A second experiment was conducted in 2020, during which growth differences among ♀K×♂K, ♀K×♂G, and female koi carp×‘F_2_ male koi×goldfish hybrid’ (♀K×♂H) backcross were investigated from 77 to 274 dpf.

Phase one of the first experiment was set up as a completely randomized design in which the factor of investigation was the cross conducted during fertilization with two levels, ♀K×♂K and ♀K×♂G. Hatched larvae were stocked (*n*=160) in duplicate in 6-liter containers in a novel static rearing system (Delomas and Dabrowski, 2018). Feeding began at 5 dpf, initially with the live rotifer *Brachionus plicatilis* and then with a combination of rotifers and newly hatched brine shrimp *Artemia salina* nauplii. After the first week of feeding, the density in each experimental unit (EU) was cut to 100 fish per container. At the conclusion of the live food period (19 dpf), all surviving fish from each tank were transferred to 10-liter tanks in a recirculating aquaculture system (RAS) (Fisher et al., 2021). Each tank was equipped with individual aeration to maintain an adequate supply of oxygen. The RAS was also equipped with both mechanical- and bio-filtration as well as ultraviolet sterilization. The fish were co-fed and then weaned to a commercial diet from 20 to 33 dpf. The fish were fed Otohime B2 (Reed Mariculture, Inc., Campbell, CA, USA) diet to apparent satiation four times per day during weaning. The number of remaining fish from each tank was further cut to 30 fish per tank at 33 dpf to decrease the density effects on the growth response (Feldlite and Milstein, 1999). The fish were then transitioned to Otohime S1 and fed at a rate of 7% biomass per day until 47 dpf.

At the beginning of phase two of the first experiment, all the fish from the ♀K×♂K tanks were removed from the replicated experimental RAS system and stocked in a 3000 liters tank in a separate RAS. Density of the remaining tanks of ♀K×♂G were further cut to 15 fish per tank by splitting the surviving fish into two tanks. The fish were fed Otohime S1 at a rate of 7% biomass per day until 65 dpf, at which point they were transitioned to Otohime S2 at 4% biomass per day until 84 dpf. Three fish from each tank of ♀K×♂G hybrids were PIT tagged at 84 dpf and combined into a single 1000 liters tank, which also included other various ♀K×♂G hybrid progenies (for which the data are not presented in this paper). After 84 dpf, both tanks of fish were fed to apparent satiation three times daily with Koi Color (3.5 mm; Skretting, Tooele, UT, USA). The tanks with ♀K×♂K and ♀K×♂G were housed in the same RAS but under different environmental conditions. The temperature (Fig. S4) and water quality of each tank were recorded. Water quality measurements (mean±SD) were recorded for ♀K×♂K and ♀K×♂G during this period of the experiment for dissolved oxygen (71.8±8.56% and 60.2±10.17%; 6.05±0.69 and 4.80±0.83 mg l^−1^) and pH (7.04±0.41 and 6.79±0.43), respectively. These values were within the normal physiological tolerances for cyprinids. The growth of each group of fish (*n*= 30–40) was monitored for mean weight over 1 year (Fig. S3).

The second experiment also utilized a completely randomized design. The factor of investigation for the experiment was the cross that was conducted during fertilization with three levels, ♀K×♂K, ♀K×♂G, and ♀K×♂H. At 77 dpf, fish with identical rearing histories for each factor were stocked (*n*=13–14 fish/tank) in triplicate in 10-liter tanks in a RAS (same as described above). A fin clip of *n*=10 fish from each cross were sampled prior to stocking to assess ploidy of individuals, these fish were not stocked in the experiment. Ploidy was assessed by flow cytometry using a procedure like that previously described by Delomas and Dabrowski (2016). Except that fin clips from individual fish were utilized, and 1.5 ml microcentrifuge tubes containing 800 μl staining solution (50 mg/l Propidium Iodide, 10 mg/l RNase A in Isoton II) with 1 μl of a 0.2×dilution of yellow perch *Perca flavascens* red blood cells in Isoton II as an internal standard. Flow cytometry samples were analyzed on a Attune Nxt Flow Cytometer (Thermo Fisher Scientific, Waltham, MA, USA) and a minimum of 10,000 gated cells were collected for each sample. The biomass stocked into each tank was measured at the beginning of the experiment. The fish in each tank were fed a commercial diet (Otohime S1 followed by S2; Reed Mariculture, Inc., Campbell, CA, USA) at the same percentage of biomass per day, which decreased from 5 to 2% during the first phase (77–147 dpf) of the experiment. At 147 dpf, four fish from each EU were injected with a passive integrated transponder (PIT) tag to enable individual identification (*n*=12 fish per cross). PIT-tagged fish were combined into a single 600-l circular tank in a common garden setup for the grow-out phase of the experiment and fed at 1% per day during the grow-out phase (148–274 dpf). Even though the fish were combined in a single common garden, the fish were treated as being from their original replicate during the sample period.

### Sampling

In phase one of the first experiment, mortalities were recorded daily, and subsamples for mean weight and total length were taken weekly and bi-weekly during the live zooplankton and commercial diet feeding periods, respectively. Subsamples (*n*= 10–20) of individual fish weight and total length were taken from each tank and were averaged for statistical analysis, with one value for each EU. At 84 dpf in the first experiment, four fish from each cross were randomly sampled and killed by an overdose of MS-222 solution (500 mg l^−1^). Samples of the brain, hepatopancreas, white muscle and red muscle were taken and immediately immersed in RNAlater (Sigma-Aldrich, St. Louis, MO, USA) at 4°C overnight before being frozen at −80°C until processing.

During the first phase of the second experiment, fish were subsampled (*n*=8 fish per EU) at 115 and 147 dpf to record the total length and weight of the individual fish. The remaining fish in each tank were bulk weighed to obtain their biomass and for adjustment of the feeding rates. The FCR was calculated as feed intake divided by weight gained for the entire first phase of the experiment. Final samples of all remaining fish were collected at the end of the grow-out phase of the experiment (274 dpf) for weight and total length.

### RNA extraction and quantification of gene expression by analyzing mRNA

The samples collected and preserved in RNAlater (Sigma-Aldrich, St. Louis, MO, USA) during fish sampling (four fish from each group×four organs, i.e. brain, hepatopancreas, white muscle and red muscle) were transferred to the laboratory of Food Sciences and Fisheries (Szczecin, Poland), where all the molecular procedures were carried out. The brain, hepatopancreas, white muscle, and red muscle samples were removed from the stabilization solution and homogenized in 750 μL of TRI Reagent^®^ (Zymo Research, USA) with 1.5/2.8 mm ceramic beads at 5000 rpm for 60 s using a Minilys^®^ personal homogenizer (Bertin Corp., USA). After centrifuging at 13.4x ***g*** for 2 min at 4°C to remove debris, the supernatant was collected, and total RNA was extracted using a Direct-zol^®^ RNA MiniPrep Kit (Zymo Research, Irvine, USA). An additional step of DNase I treatment was applied to avoid genomic DNA contamination, according to the instructions recommended by the manufacturer. The quantity and quality of the RNA were assessed using a NanoDrop 2000 (Thermo Fisher Scientific, Waltham, USA) and via electrophoresis on a 2% agarose gel. The 260/280 ratio of all RNA extracts was approximately 2.0, and no signs of RNA degradation were observed. Next, RNA extracts were equalized to concentration of 500 ng/µl and used in cDNA synthesis. Reverse transcription was performed using the Transcriptor First Strand cDNA Synthesis Kit (Roche, Basel, Switzerland) according to the manufacturer's instructions with Anchored oligo(dT)18 primers. To assess expression of *igf-1*, *adh* and *pdh E1β_2_* in fish samples following procedure was applied. First, primer pairs were designed based on the sequences retrieved from GeneBank using default parameters in Primer3 software (Table 1) (Koressaar and Remm, 2007). Next, three decimal dilutions of synthesized cDNA were performed to assess qPCR efficiency for each primer pair (Table 1). All reactions were performed on a LightCycler^®^ 480 II (Roche, Basel, Switzerland) using LightCycler^®^ FastStart DNA Master SYBR Green (Roche, Basel, Switzerland), 0.1 µM of each primer and 1 µl of cDNA template in a final volume of 20 µl, in duplicates. Cycling conditions included an initial 10 min of activation at 95°C, followed by 40 cycles of denaturation at 95°C for 10 s and annealing at 60°C for 10 s and extension at 72°C for 15 s. Additionally, melting curve analysis (65–95°C) was conducted at the end of each PCR thermal profile to ensure the specificity of the amplification.

**Table 1.**
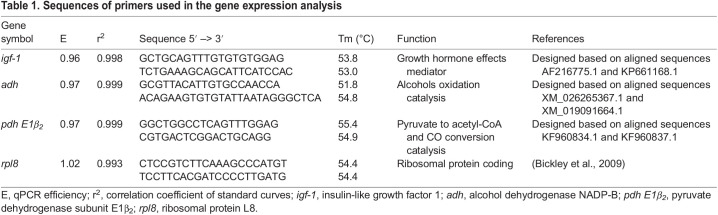
Sequences of primers used in the gene expression analysis

After finalizing all initial steps, real-time PCR were performed for *igf-1*, *adh* and *pdh E1β_2_*, and the expression of the tested genes were normalized against an internal reference, the 60S ribosomal protein L8 gene (*rpl8*), which was chosen based on previous studies on cyprinids (Bickley et al., 2009; Eljasik et al., 2020). Relative gene expression was calculated using the 2^−ΔΔCt^ method (Livak and Schmittgen, 2001) in GeneEx (MultiD Analyzes, Göteborg, Sweden) software, and the data are presented on a linear scale. Statistical analysis of the qPCR results was made based on ΔCt results (log transformed 2^−ΔΔCt^).

### Statistical analysis

A general linear mixed model was fitted to the response variables of total length and weight in both experiments one and two. The linear predictors of the statistical model included the fixed effects of cross, age (dpf), and their two-way interaction. Repeated measures over time were modeled using an ante-dependence covariance structure of the first order in the first experiment and using an unstructured covariance structure for the second experiment fitted to observations from each tank nested within cross. These covariance structures were selected as the best fit from a subset of plausible structures based on the Akaike information criterion (AIC). Variance components were estimated using restricted maximum likelihood. Notably, for the model of weight in the first experiment to fully estimate variance components, a log_10_ transformation of the data was needed. Kenward Roger's method was used to estimate degrees of freedom and make corresponding adjustments in the estimation of standard errors. Model assumptions were evaluated using externally studentized residuals. The estimated least square means (lsmeans) and corresponding estimated standard errors are presented (except for the experiment one weight results, which are presented as 95% confidence intervals due to back transformation of the data). Pairwise comparisons of marginal means and cell means were conducted using Tukey–Kramer's and Bonferroni's adjustment, respectively, for multiple comparisons to prevent inflation of Type I error. Models were fitted using the Glimmix procedure of SAS (version 9.4; SAS Institute, Cary, NC, USA), and all the results were considered significant at α=0.05.

A general linear model was fitted to the response variable of FCR. The linear predictor of the statistical model included the fixed effect of cross. Model assumptions, variance components, and estimations of degrees of freedom were conducted as previously described. The equal variance assumption was met. However, the normality of the residuals was not. Different data transformations, including the square-root, log_10_, ln, and squared (x^2^) transformations, were assessed, but all the transformations did not improve the normality of the residuals and often exacerbated it. The model was fitted to non-transformed data since previous studies have shown that a one-way ANOVA is generally robust to violations of this assumption (Schmider et al., 2010).

Student’s *t*-tests were conducted to compare the relative expression of each gene in the sampled tissues between ♀K×♂K and ♀K×♂G, and the results were considered significant at α=0.05. Except for the *igf-1* in red muscle, which had unequal variances requiring a Welch's *t*-test to be used instead. Additionally, the normality assumption for *igf-1* in brain was failed, and a non-parametric Mann–Whitney U test was used.

## Supplementary Material

10.1242/biolopen.060342_sup1 Supplementary information
