# Supplementary material for: Inherited anoxia tolerance and growth performance can result in enhanced invasiveness in hybrid fish
Source: Biol Open. 2024 Oct 21;13(10):bio060342. doi: 10.1242/bio.060342 (PMC11554265; doi:10.1242/bio.060342)
Supplement: Supplementary information [file biolopen-13-060342-s1.pdf]

Biology Open (2024): doi:10.1242/bio.060342: Supplementary information

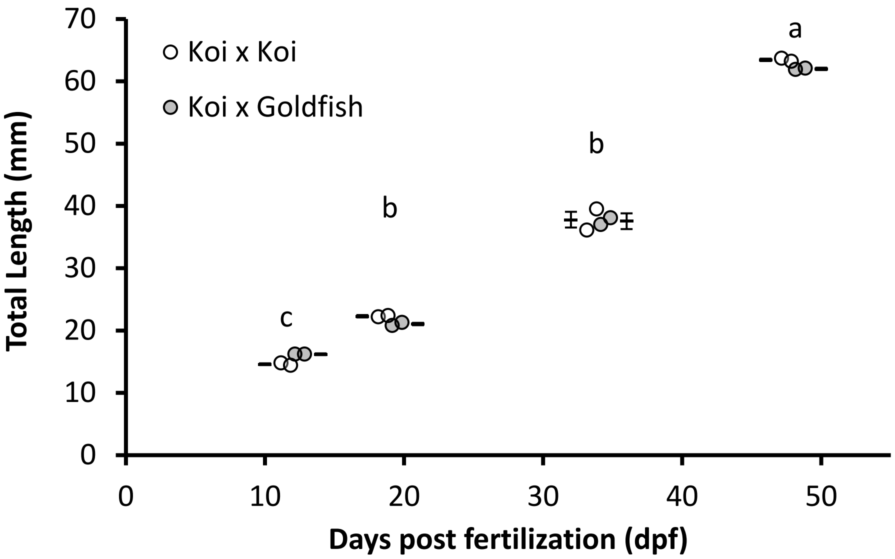

Fig. S1. Total length (mm) of koi × koi (♀K×♂K) and koi × goldfish (♀K×♂G) hybrids over the course of experiment one (12-48 dpf). The data points represent individual replicate means (n=2) obtained from subsamples of n=10 fish per replicate with lsmeans ± SE presented. Repeated measurements analysis revealed a significant main effect of age (p=0.0033). Tukey's adjustment was used and ages (dpf) not sharing common letters are significantly different.

Biology Open • Supplementary information

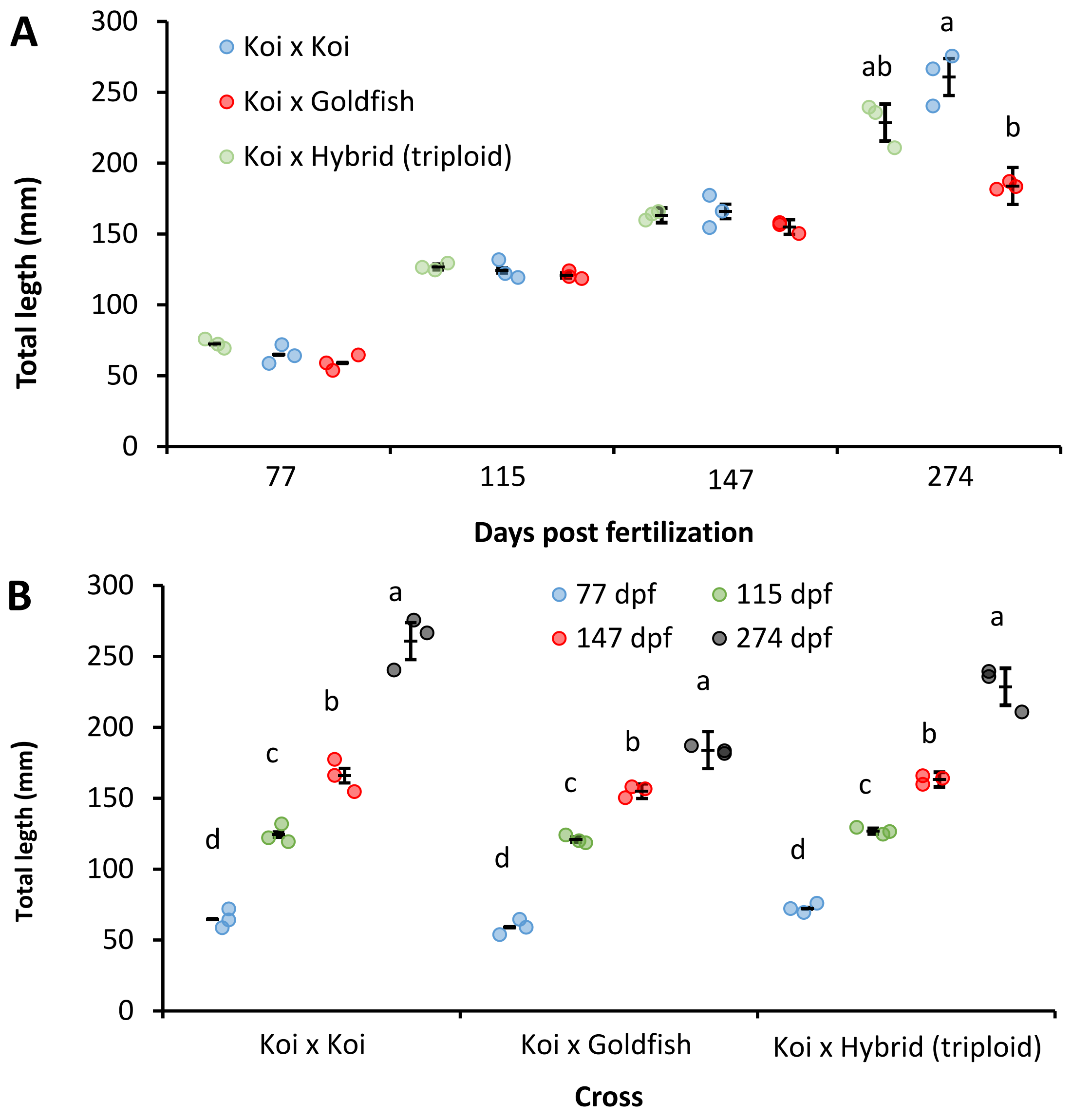

Fig. S2. Total length (mm) of koi × koi (♀K×♂K), koi × goldfish (♀K×♂G), and koi × hybrid (♀K×♂H) hybrids over the course of experiment two (77-274 dpf). The data points represent replicate means (n=3) obtained from subsamples of n=10 fish per replicate, and the bars are lsmeans ± SE. Repeated measurements analysis revealed a significant interaction effect between cross and age (dpf) ($p=0.0339$). A) Simple effects of cross within levels of age (dpf) are presented at the top, and B) simple effects of age (dpf) within levels of cross are presented at the bottom. A Bonferroni adjustment was utilized for multiple comparisons with levels not sharing a common letter being significantly different ($p < 0.05$).

Biology Open • Supplementary information

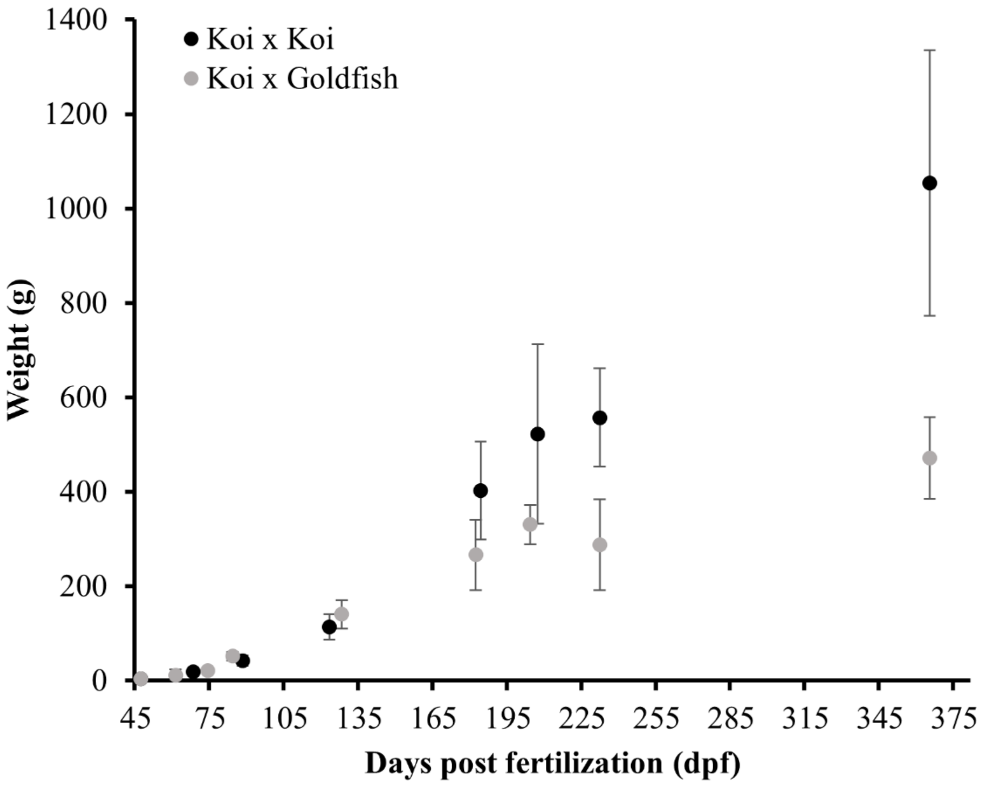

Fig. S3. Growth (mean ± SD) of koi × koi and koi carp × goldfish from 47 dpf to 365 dpf in experiment 1.

Biology Open • Supplementary information

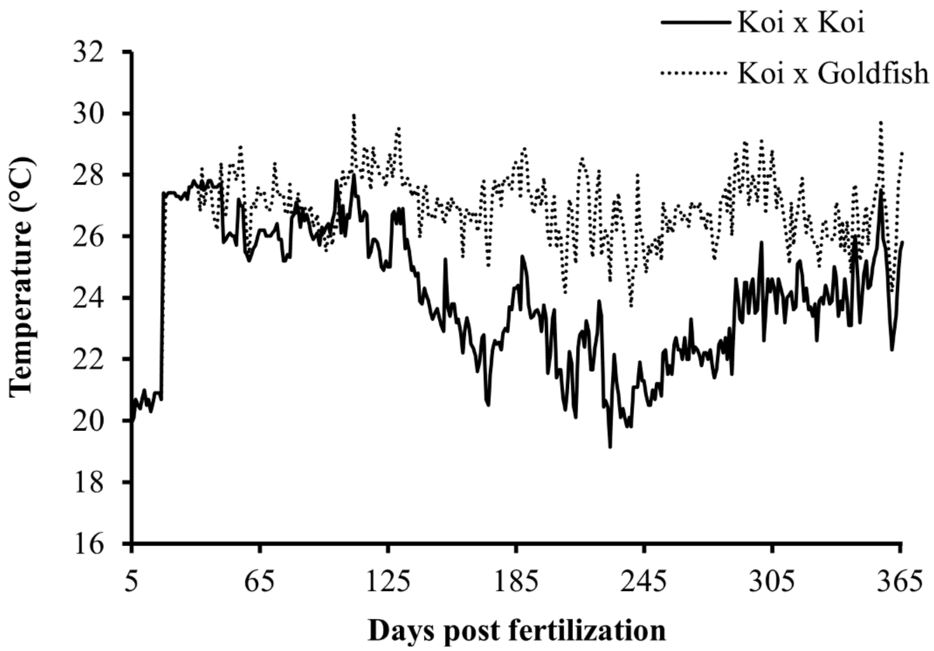

Fig. S4. Water temperature during the first year of life for koi × koi and koi × goldfish hybrids throughout the first experiment.

Biology Open • Supplementary information
